# The Differential Diagnosis of Uterine Hæmorrhages.—I

**Published:** 1910-01-22

**Authors:** 


### Gynæcology.

### THE DIFFERENTIAL DIAGNOSIS OF UTERINE HÆMORRHAGES.—I.

Hæmorrhage from the uterus has to be considered from the point of view of the pregnant and the unimpregnated condition of the organ. The presence of pregnancy can never be assumed, but must be clearly established by a general survey of all the signs and symptoms of the case. For our present purpose it will be assumed that there is no doubt that a pregnancy exists, and that some hæmorrhage has occurred. In the early months it has long been believed that menstruation may occur for one or more periods up to the time when the placenta is fully formed and the uterine cavity obliterated. Although it must be admitted that occasionally a hæmorrhage occurs which comes on at the exact time of a period and lasts the usual time, yet in the majority of cases this exact periodicity is not, in fact, established if the history is carefully gone into. Further, when more than one so-called period occurs careful scrutiny usually reveals that there is no exact periodicity. This leads inevitably to the belief that these so-called periods are not really menstrual at all, but are, in fact, evidence of the separation of some part of the embryo with bleeding, and indicate an attempt at abortion. Those patients who state that they menstruate all through pregnancy are more pronounced examples of this condition, and must be looked upon always as cases of threatened abortion or premature labour with hæmorrhage. It must be accepted in the light of our present knowledge that a person who is really pregnant ceases to menstruate, and that any hæmorrhage which occurs represents some condition or lesion which is not really menstruation. The converse is also true, namely, that a person who menstruates regularly and typically cannot be pregnant. These cases can very often be further made clear by the presence of some pain during the period of hæmorrhage, the pain usually coinciding with uterine contractions and being further evidence of threatened abortion. Such pain, however, is not always due to contractions, but may be the result of intra-uterine clotting of blood, leading to some stretching of the uterine wall and non-contraction of the adjacent part of the uterine muscle. These clots will show themselves when delivery finally occurs as old more or less organised structures.

Any hæmorrhage, then, in the early months of pregnancy, accompanied or not by pain, must be looked upon as evidence of a threatened abortion and treated as such. The bleeding caused by a hydatidiform mole is fairly characteristic, and is more of the nature of a continuous watery blood-stained discharge with occasional excessive floodings. This discharge has been compared from time immemorial to red-currant juice. The hæmorrhage of a threatened abortion is rarely continuous for long, but comes and goes with rest and treatment, whilst that of a vesicular mole is usually continuous. Further, the uterus itself is a great aid to diagnosis in such cases, for it is nearly twice as large as it should be for the period of amenorrhœa, usually reaching the umbilicus at the third month. The consistence of the uterus too is suggestive; it is doughy in character, and sometimes shows areas of irregular contraction, whilst other parts are relaxed. The appearance of a hydatidiform vesicle in the discharge makes the diagnosis certain. The hæmorrhage of a carneous mole is not characteristic, and will be often mistaken for a simple threatened abortion, but if the mole has existed any time the uterus will be smaller than the period of amenorrhœa would seem to suggest it should be. Sometimes, too, owing to infection and subsequent sapræmia, the hæmorrhage is accompanied by foul discharge. Carneous moles are usually expelled spontaneously, and rarely call for treatment unless bleeding becomes excessive and demands emptying of the uterus.

Incomplete abortion gives rise to hæmorrhage which is often continuous with occasional exacerbations, "floodings." The diagnosis may be exceedingly difficult unless something which has been expelled from the uterus has been seen and recognised as a product of conception. More usually these cases are not seen at the time of the abortion and only come for treatment when hæmorrhage continues. Then there always arises the difficulty of establishing the diagnosis of pregnancy. In such a case the history must be carefully scrutinised; if amenorrhœa has occurred with other signs, such as morning sickness and breast changes, the presence of a pregnancy may be inferred, and the history of the passage of blood and clots with pain will then make abortion at least presumptive. As a rule in incomplete abortion the uterus is only a little larger than normal, and the cervix is generally closed to the examining finger. This last, however, depends on the length of time which has elapsed since the abortion. In all these cases of bleeding in the early months of pregnancy the presence of backward displacements and the history of previous menorrhagia with leucorrhœa and backache make fairly certain the presence of uterine congestion and endometritis.

Although threatened abortion is the commonest cause of bleeding in early pregnancy, it must not be forgotten that there are occasional circumstances which may set up bleeding apart from pregnancy itself in a pregnant woman. Foremost among these are carcinoma of the cervix, mucous polypi of the cervix, and occasionally cervical erosion. Sometimes slight bleeding may occur from urethral caruncle or other non-uterine lesion. It is the friability of a carcinoma which is its distinguishing character, combined with hardness and induration. It is, however, a friable hardness, and not the tough leathery hardness which is the distinguishing feature of the chronic inflammatory conditions which lead to cervical erosion. Polypi of the cervix are apt to become exceedingly vascular in pregnancy and bleed on the slightest touch; movements during defæcation and micturition may be sufficient to start them bleeding, just as these movements will cause a cancer to bleed. Urethral caruncle is sufficiently obvious on inspection of the vulva.

